# Supplementary material for: Regulation of tumor angiogenesis and mesenchymal–endothelial transition by p38α through TGF-β and JNK signaling
Source: Nat Commun. 2019 Jul 11;10:3071. doi: 10.1038/s41467-019-10946-y (PMC6624205; doi:10.1038/s41467-019-10946-y)
Supplement: Supplementary file 1 — Supplementary information [file 41467_2019_10946_MOESM1_ESM.pdf]

# SUPPLEMENTARY INFORMATION

## Regulation of tumor angiogenesis and mesenchymal-endothelial transition by p38α through TGF-β and JNK signaling

Raquel Batlle[1], Eva Andrés[1], Lorena Gonzalez[1], Elisabet Llonch[1], Ana Igea[1], Núria Gutierrez-Prat[1], Antoni Berenguer-Llergo[1] and Angel R. Nebreda[1,2,*]

[1]Institute for Research in Biomedicine (IRB Barcelona), The Barcelona Institute of Science and Technology, 08028 Barcelona, Spain
[2]ICREA, Pg. Lluís Companys 23, 08010 Barcelona, Spain
*email: angel.nebreda@irbbarcelona.org

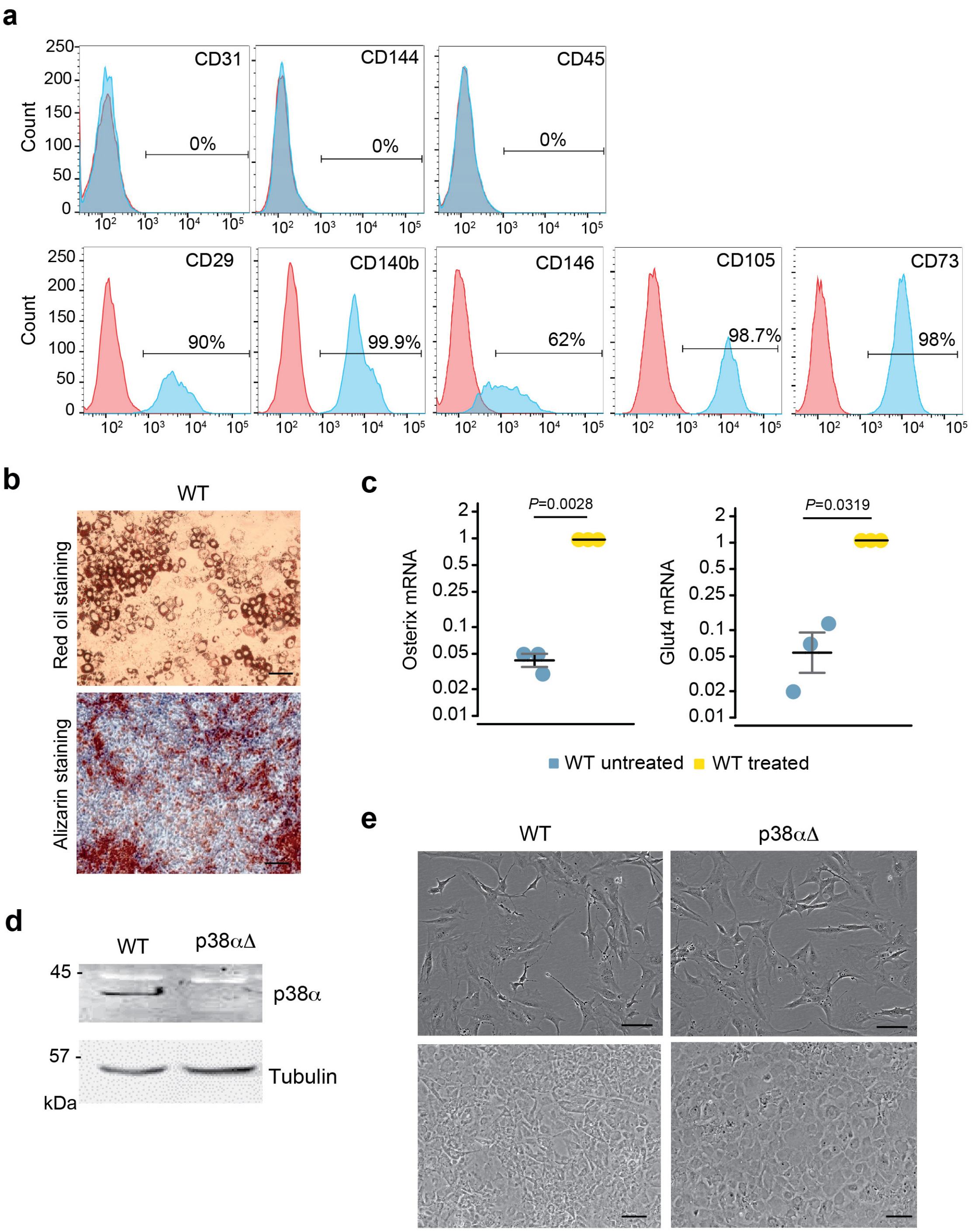


**Supplementary Fig. 1.** Characterization of MSCs. **a,** Bone marrow MSCs were isolated from femurs and tibiae of *Mapk14*$^{lox/lox}$;UBC-Cre-ERT2 mice, immortalized and stained with the indicated antibodies (blue) or with isotype-matched control immunoglobulins (red), and were analyzed in a FACS Aria 2.0. **b,** WT MSCs were cultured in osteoblast or adipocyte differentiation media. Culture medium was replaced by DMEM with 10% FBS, and 2 days later cells were stained with either Oil Red (adipocytes) or Alizarin Red (osteoblast). Bars, 100 µm. **c,** Relative mRNA expression levels for the adipocyte marker GLUT4 and the osteoblast marker Osterix were determined by qRT-PCR in both untreated cells and at the end of the differentiation protocol. Values are graphically represented in log2 scale and show the fold change versus WT untreated. Data are mean

± SEM (n=3). **d,** MSCs were treated for 2 days with 4-OHT to obtain p38αΔ MSCs. WT MSCs were treated with vehicle. The expression of p38α was evaluated by immunoblotting using tubulin as a loading control. **e,** Phase contrast light micrographs of subconfluent (upper panels) and confluent (lower panels) MSCs cultured with 0.5% FBS. Note that non-confluent WT and p38αΔ MSCs have long thin cell bodies with a large nucleus, whereas confluent WT and p38αΔ MSCs show the spindle and cobblestone patterns, respectively. Bars, 100 μm.

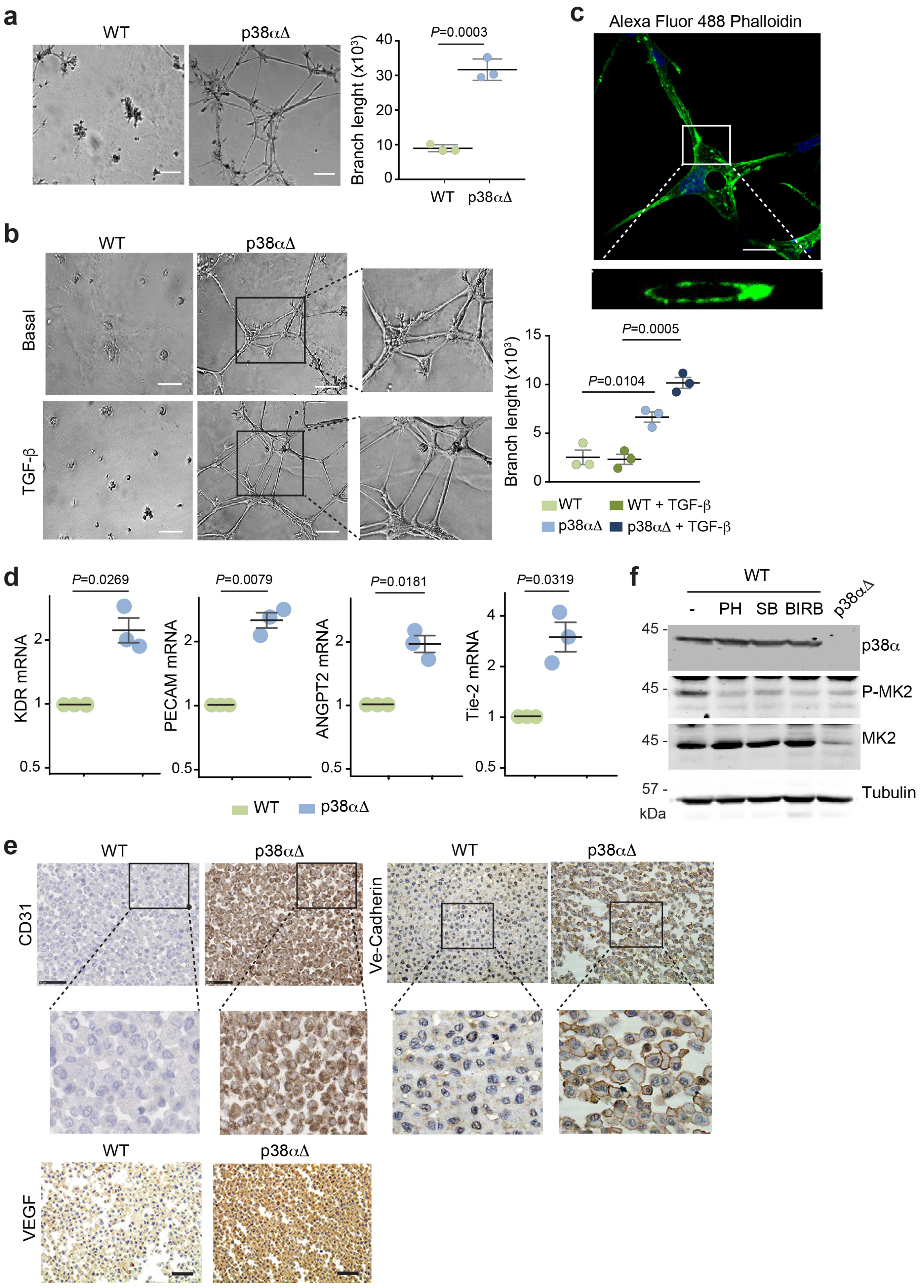


**Supplementary Fig. 2.** p38α regulates the formation of capillary-like structures by MSCs *in vitro.* **a,** Primary MSCs were isolated from femurs and tibiae of *Mapk14*$^{lox/lox}$;UBC-Cre-ERT2 mice, and were treated for 2 days with 4-OHT to obtain p38αΔ MSCs. WT MSCs were treated with vehicle. WT and p38αΔ MSCs were seeded in matrigel with 0.5% FBS for 16 h. The histogram shows the quantification of the branch length using ImageJ. Data are mean ± SEM (n=3). Bar, 100 µm. **b,** MSCs were

cultured in collagen with 0.5% FBS in the presence or absence of TGF-β (5 ng/ml), and tube formation was determined two days later. Bars, 100 μm. The right panels show higher magnifications of the indicated areas. The histogram shows the quantification of the branch length using ImageJ. Data are mean ± SEM (n=3). **c,** Primary p38αΔ MSCs were cultured in a 3D collagen gel to form vascular structures and were stained with Alexa Fluor 488 Phalloidin. The lower panel shows a higher magnification of the indicated area as the orthogonal projections along the vertical axis of the central lumen. Bar, 25 μm. **d,** Relative mRNA levels for the indicated endothelial markers in WT and p38αΔ MSCs. Values were graphically represented in log2 scale and show the fold change versus WT. Data shown are mean ± SEM (n=3). **e,** MSC pellets were analyzed by immunohistochemistry with antibodies for CD31, Ve-Cadherin and VEGF. The bottom panels show higher magnifications of the indicated areas stained for CD31 and Ve-Cadherin. Bars, 100 μm. **f,** Protein lysates were prepared from WT MSCs treated with the p38α inhibitors SB203580 (SB, 10 μM), PH787904 (PH, 1 μM) or BIRB0796 (BIRB, 200 nM), and were analyzed by immunoblotting with the indicated antibodies.

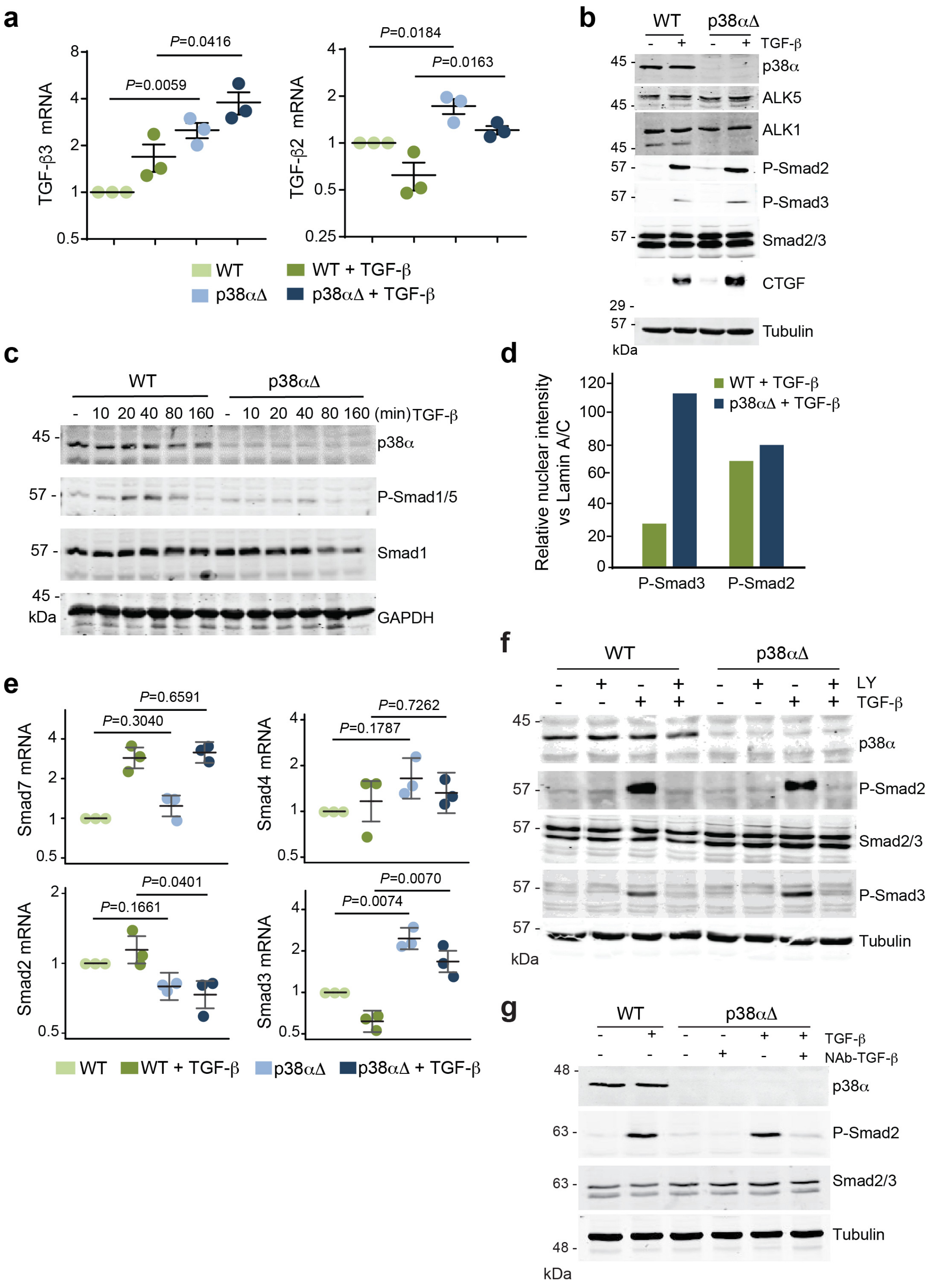


**Supplementary Fig. 3.** p38α negatively regulates Smad3 phosphorylation in MSCs. **a** and **b,** MSCs were incubated in 0.5% FBS and treated with TGF-β for 20 h. Total RNAs were purified and analyzed by RT-PCR (**a**), and protein lysates were analyzed by immunoblotting (**b**). **c,** MSCs were treated with TGF-β and collected at the indicated times followed by immunoblotting analysis**. d,** MSCs either untreated or treated with TGF-β for 20 h were analyzed by immunoblotting as shown in Figure 4g.

Quantification of the nuclear fractions is presented. **e,** Relative levels of the indicated mRNAs in WT and p38αΔ MSCs treated or not with TGF-β were analyzed by RT-PCR. Data show the mean ± SEM (n=3). **f,** Total lysates were prepared from MSCs treated with TGF-β and/or the TGF-β inhibitor LY2157299 (LY, 1 nM), and were analyzed by immunoblotting with the indicated antibodies. **g,** Lysates were prepared from MSCs treated with TGF-β and/or the TGF-β 1, 2, 3 Antibody (2 ng/mL) and were analyzed by immunoblotting with the indicated antibodies.

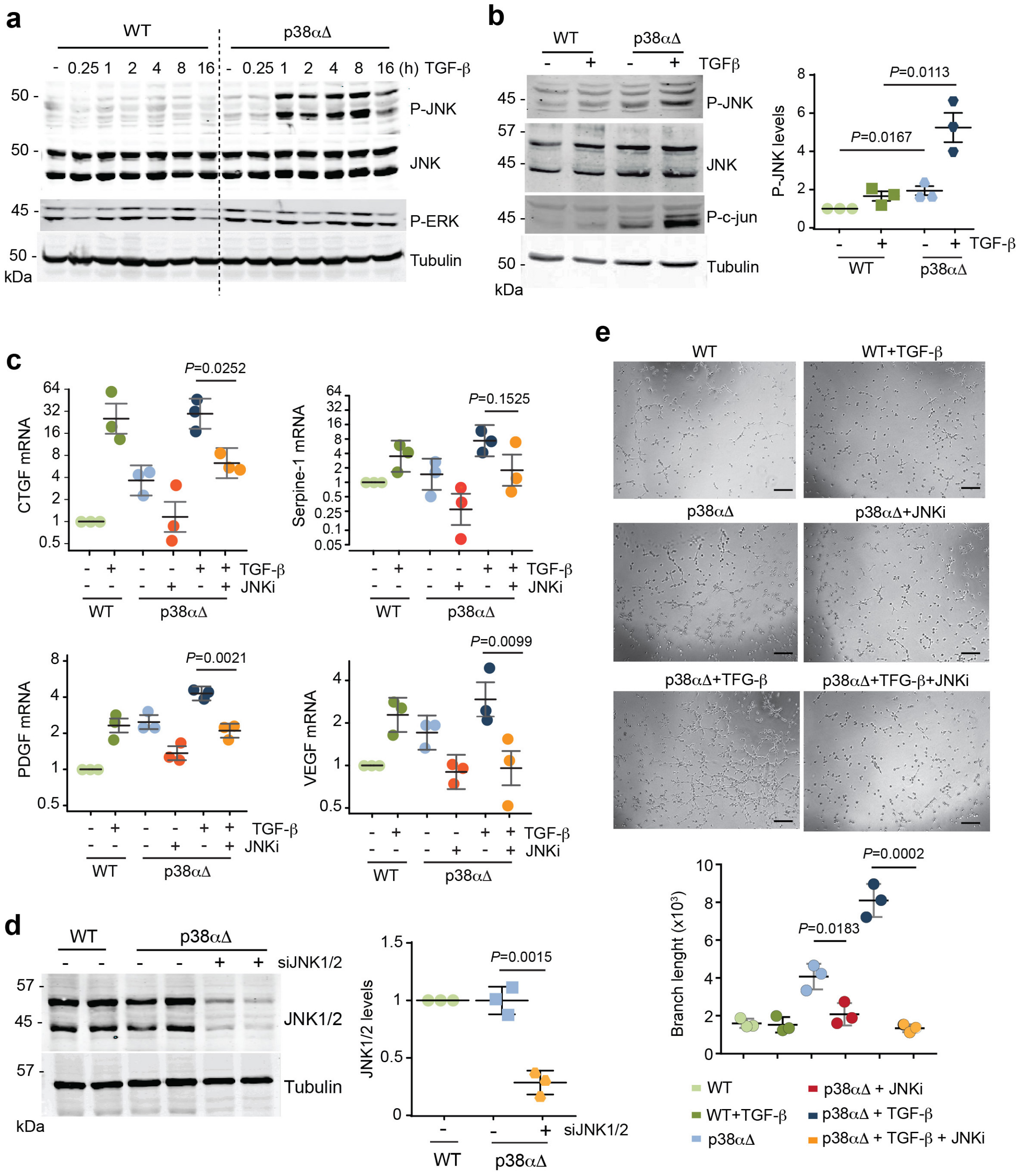


**Supplementary Fig. 4.** p38α regulates expression of TGF-β target genes though the JNK pathway. **a,** MSCs were incubated in 0.5% FBS, treated with TGF-β for the indicated times, and total cell lysates were analyzed by immunoblotting. The molecular weights indicated for P-JNK and JNK immunoblots represent pre-stained markers. **b,** MSCs were treated with TGF-β for 20 h, and were analyzed by immunoblotting with the indicated antibodies. The histogram shows the quantification of phospho-JNK levels in three independent experiment using ImageJ. Values show the fold change versus WT. Data are mean ± SEM (n=3). **c,** MSCs were cultured in 0.5% FBS for 24 h, and then TGF-β and/or the JNK inhibitor SP600126 (JNKi, 20 μM) were added to the medium sequentially with a 2 h interval. Relative levels for the indicated mRNAs were determined by qRT-PCR. Values are graphically represented in log2 scale and show the fold change versus WT. Data are mean ± SEM (n=3). **d,** Total lysates from MSCs either untreated or treated with JNK1 and JNK2 siRNAs were analyzed by immunoblotting

with the indicated antibodies. Two independent samples were analyzed in the immunoblot shown. The histogram shows the quantification of JNK1/2 levels in three independent experiment using ImageJ. Values show the fold change versus WT. Data are mean ± SEM (n=3). **e,** MSCs were starved and pre-treated with the JNK inhibitor BI-78D3 (JNKi) for 2 h and then treated with TGF-β. The following day, $1x10^4$ cells were seeded in matrigel with 0.5% FBS. TGF-β and BI-78D3 were added to the culture, and tube formation was determined 6 h later. Bars, 100 μm. Branch length per field was quantified using ImageJ. Data are mean ± SEM (n=3).

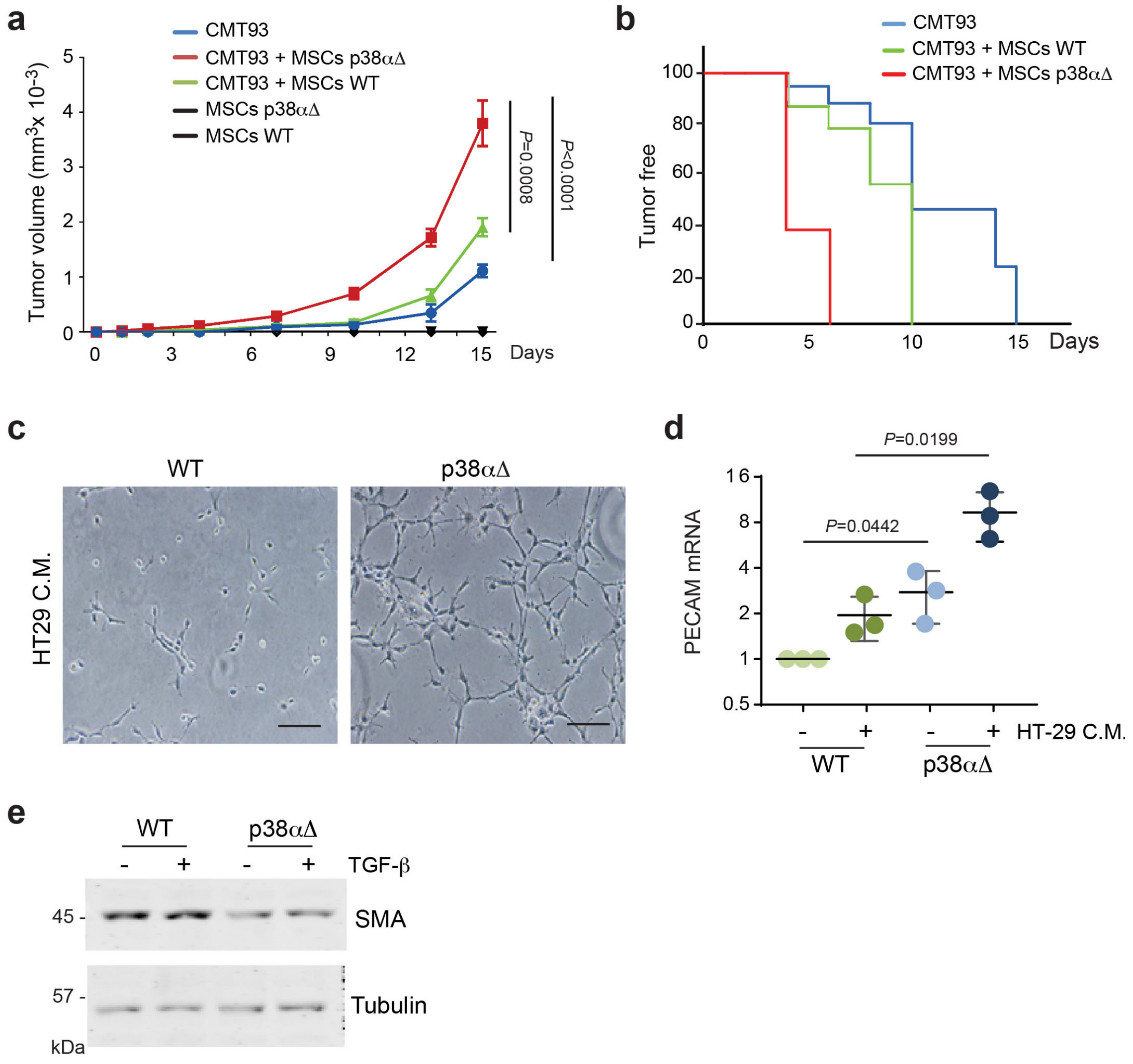


**Supplementary Fig. 5.** p38α-deficient MSCs stimulate colon cancer cell growth and angiogenesis. **a,** Growth kinetics of $2x10^6$ CMT93 colon cancer cells subcutaneously implanted into nude mice either alone or in combination with $5x10^5$ WT or p38αΔ MSCs. As a control MSCs were also implanted alone. Day 1 indicates when the tumors were detectable and started to be measured. Values are mean ± SEM (n=8). **b,** Kaplan-Meier curves for the mice in **a**. **c,** MSCs were incubated in matrigel with conditioned media (C.M.) from HT29 cells and tube formation was analyzed 6 h later. Bars, 100 µm. **d,** Relative levels for PECAM (CD31) mRNA in MSCs incubated with the conditioned media of HT29 for 16 h. Data are mean ± SEM (n=3). **e,** Total protein lysates were prepared from MSCs treated with TGF-β for 16 h, and were analyzed by immunoblotting.

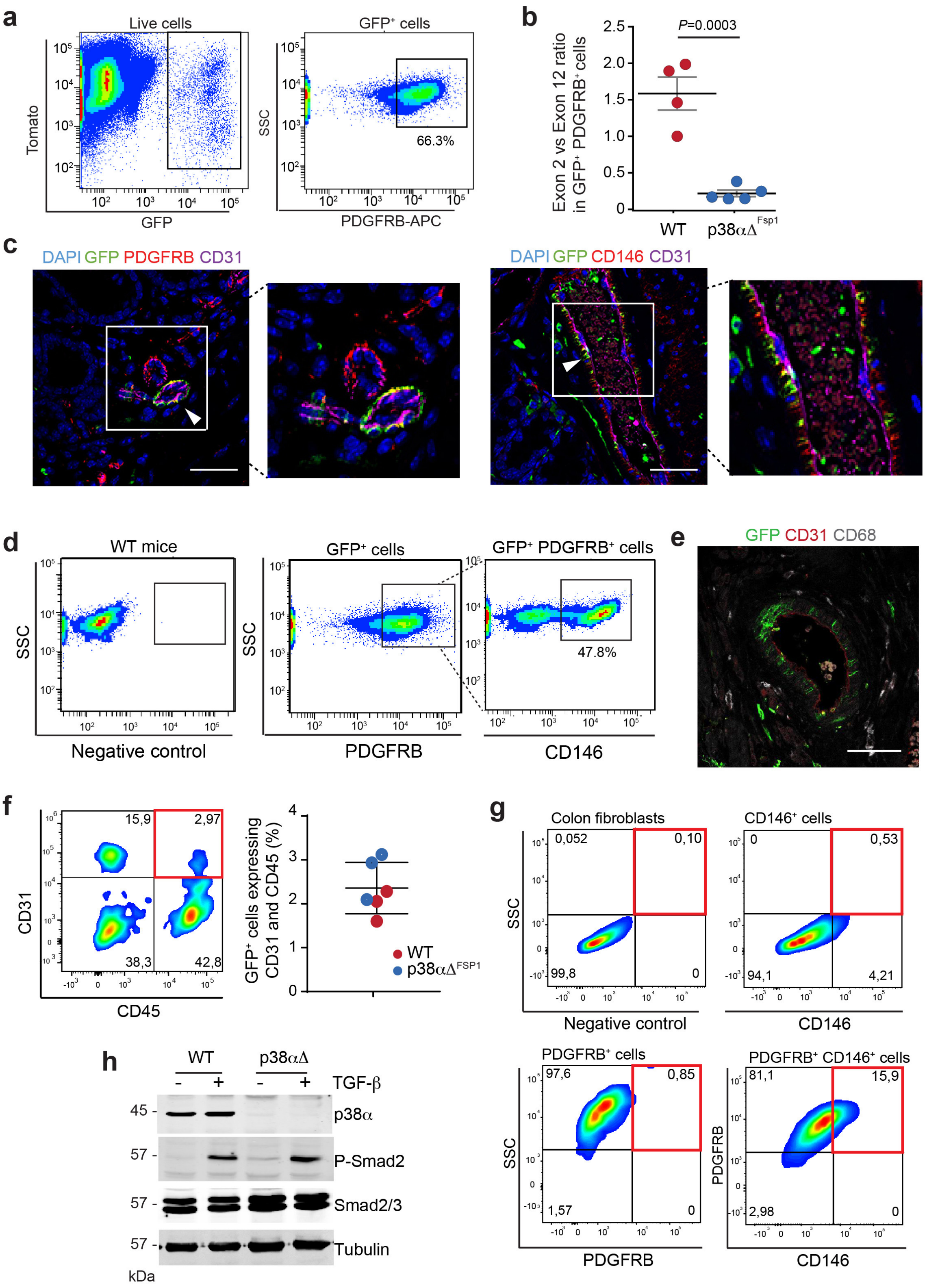


**Supplementary Fig. 6.** Characterization of FSP1-Cre expression in the colon of mice. **a,** Colons from 5 WT animals were enzymatically dissociated, and cell suspensions were directly stained with labeled antibodies and analyzed on a FACS Aria cell sorter. Analysis of GFP$^+$ cells stained with PDGFRB to identify perivascular MSCs is shown. **b,** Relative amount of the floxed exon 2 versus exon 12 (as a control) of the *Mapk14* gene was determined in GFP$^+$ PDGFRB$^+$ cells by qPCR. Data are mean ± SEM (n=4

WT, n=5 p38αΔ$^{FSP1}$ mice). **c,** Colon tissues were stained for the endothelial marker CD31 (magenta), FSP-1-driven GFP (green), and the mural markers PDGFRB or CD146 (red). GFP$^{+}$ cells reside adjacent to CD31$^{+}$ endothelial cells and co-localize with PDGFRB or CD146, indicated by a white arrowhead. Bars, 100 μm. **d,** Flow cytometry scheme indicating the analysis of GFP$^{+}$ cells in colons from FSP1-Cre/Tomato-GFP mice (n=5). About 1.1% +/- 0.68% (mean ± SEM) GFP$^{+}$ cells were detected and further stained with PDGFRB and CD146 to identify perivascular MSCs. **e,** Colon tissues from p38αΔ$^{FSP1}$ mice expressing Tomato-GFP were stained for FSP1-driven GFP (green), the endothelial marker CD31 (red) and the monocyte marker CD68 (grey). **f,** Flow cytometry analysis to quantify the percentage of GFP$^{+}$ cells that also express both CD45 and CD31 in colons from FSP1-Cre mice expressing Tomato-GFP. Data represent mean ± SEM (n=6 mice). About 2.34% +/- 0.236% (mean ± SEM) of cells were stained with CD45 and CD31. **g,** Flow cytometry scheme indicating the approach to isolate perivascular cells from colon. Cells were stained with PDGFRB and CD146 to identify perivascular cells from UBC-Cre-ERT2 mouse. **h,** Colon fibroblasts were obtained from *Mapk14*$^{lox/lox}$/UBC-Cre-ERT2 mice and PDGFRB$^{+}$ CD146$^{+}$ cells were sorted and then incubated with 4-OHT or vehicle for 2 days. Cells were treated with TGF-β for 20 h, and total cell lysates were analyzed by immunoblotting.

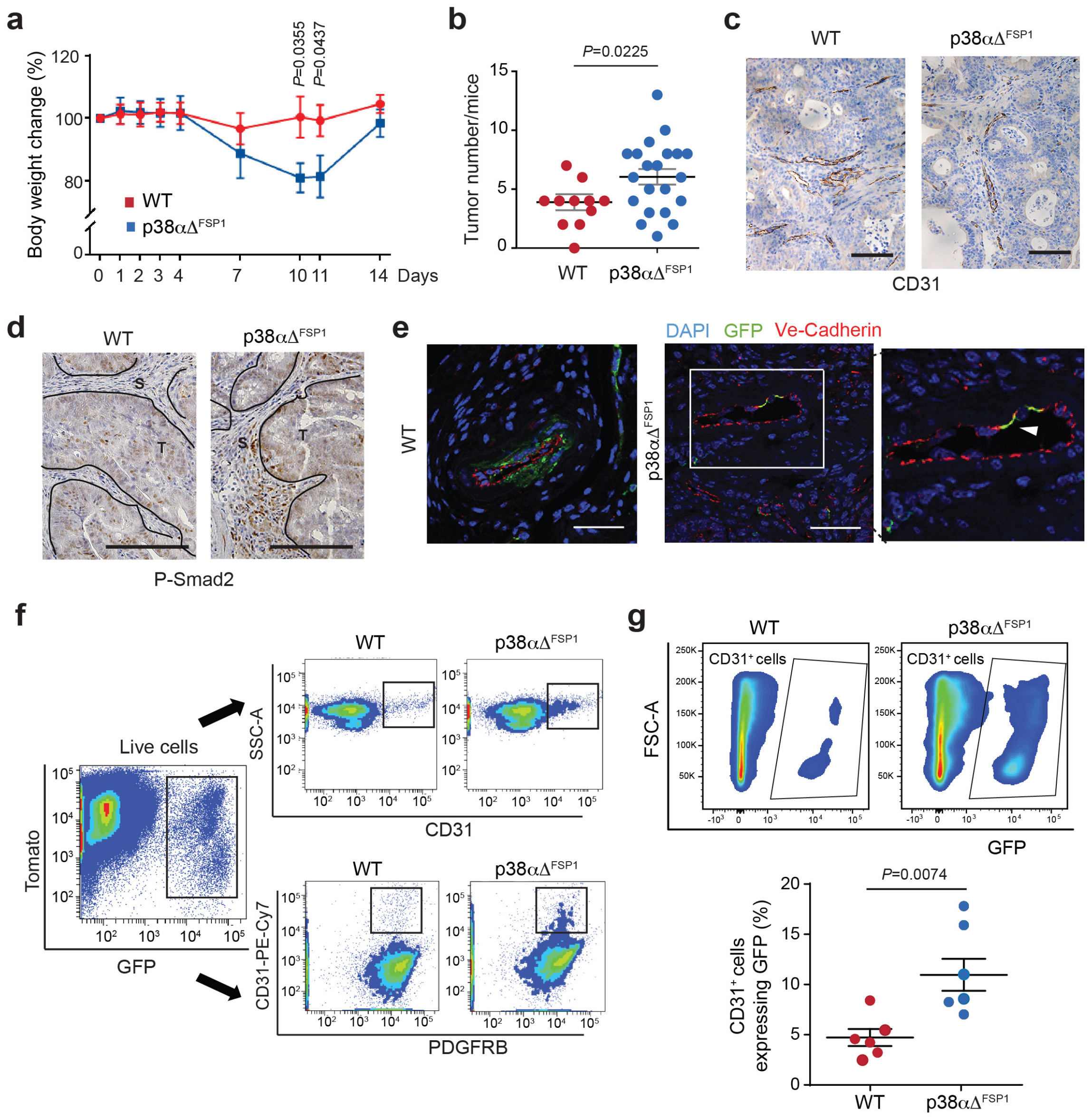


**Supplementary Fig. 7.** p38α signaling in MSCs negatively regulates tumor angiogenesis in FSP-1 mice. **a,** Body weight of WT and p38αΔ$^{FSP1}$ mice injected with AOM and treated with one DSS cycle. Data are mean ± SEM (n=22 WT, n=39 p38αΔ$^{FSP1}$). **b,** Total number of tumors formed in WT and p38αΔ$^{FSP1}$ mice at the end of the AOM/DSS protocol. Data represent mean ± SEM (n=11 WT, n=21 p38αΔ$^{FSP1}$). **c,** Colon sections from WT and p38αΔ$^{FSP1}$ mice with Tomato-GFP were stained with CD31 antibody. Bars, 100 μm. **d,** Phospho-Smad2 staining of colon tumors obtained in WT and p38αΔ$^{FSP1}$ mice treated with AOM/DSS. T, tumor; S, stroma. Bars, 100 μm. **e,** Colons from non-treated WT and p38αΔ$^{FSP1}$ Tomato-GFP mice were immunostained with antibodies for GFP (green) and the endothelial marker Ve-Cadherin (red). Co-staining is indicated by a white arrowhead. Bars, 100 μm. The right panel shows a higher magnification of the indicated area. **f,** Flow cytometry scheme for the analysis of GFP$^{+}$ cells obtained from FSP1-Cre mice and stained with PDGFRB for perivascular cells and CD31 for endothelial cells. **g**, Flow cytometry analysis to quantify the percentage of CD31$^{+}$ cells that also express GFP in colon tumors from FSP1-Cre mice expressing Tomato-GFP and treated with AOM/DSS. Data represent mean ± SEM, n = 6.

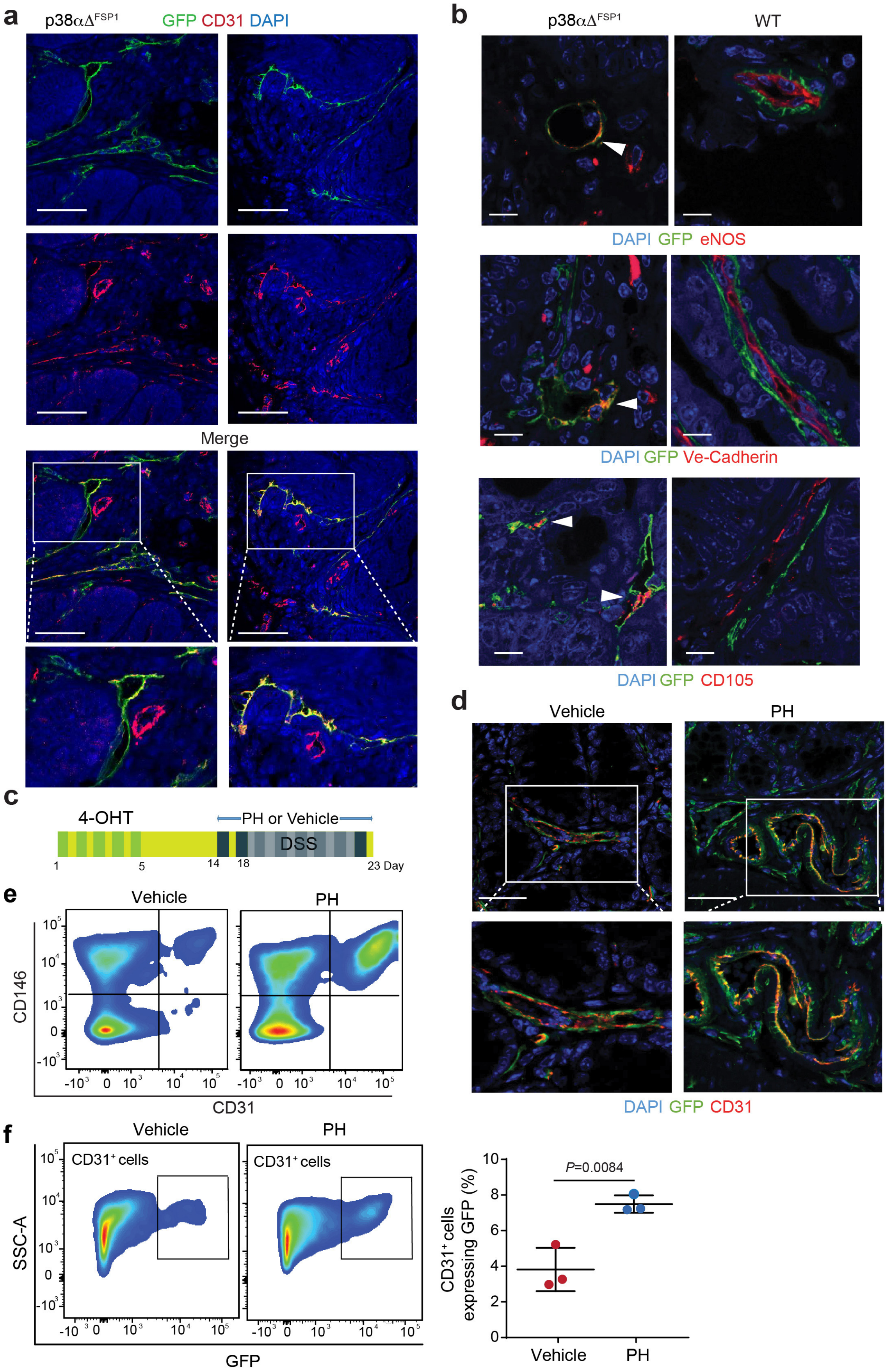
a
p38αΔFSP1
GFP CD31 DAPI
Merge
b
p38αΔFSP1
WT
DAPI GFP eNOS
DAPI GFP Ve-Cadherin
DAPI GFP CD105
c
4-OHT
PH or Vehicle
DSS
1
5
14
18
23 Day
d
Vehicle
PH
DAPI GFP CD31
e
Vehicle
PH
CD146
CD31
f
Vehicle
PH
CD31+ cells
SSC-A
GFP
P=0.0084
CD31+ cells expressing GFP (%)
Vehicle
PH

**Supplementary Fig. 8.** p38α negatively regulates endothelial marker expression in PDGFRB$^{+}$ mesenchymal cells from FSP1-Cre and PDGFRB-Cre mice. **a,** Colon tumors from p38αΔFSP1 mice expressing Tomato-GFP were immunostained with antibodies for GFP (green) and the endothelial marker CD31 (red). Co-staining is indicated by white arrowheads. Bar, 100 μm. **b,** Colon tumors from WT and p38α$\Delta^{FSP1}$ Tomato-GFP mice were immunostained with antibodies for GFP (green) and the endothelial markers CD105, VE-Cadherin or eNOS (red). White arrowheads indicate co-staining. Bars, 25 μm. **c,** Schematic representation of the DSS protocol. **d,** PDGFRB-Cre-ERT2 mice expressing Tomato-GFP were treated with 4-OHT and then with the p38α inhibitor PH797804 (PH) or vehicle. Colons were immunostained with antibodies for GFP (green) and the endothelial markers CD31, Bars, 100 μm. **e,** Flow cytometry scheme for the analysis of GFP$^{+}$ cells obtained from colons of PDGFRB-Cre-ERT2/Tomato-GFP mice treated with DSS and either PH or vehicle, and stained with CD146 for perivascular cells and CD31 for endothelial cells. **f,** Flow cytometry analysis to quantify the percentage of CD31$^{+}$ cells that also express GFP in colons from PDGFRB-Cre-ERT2/Tomato-GFP mice treated with DSS and either PH or vehicle. Data represent mean ± SEM, n=3.

.

**Supplementary Table 1.** Antibodies used for FACS analysis

| | Dilution | Provider |
|---|---|---|
| CD31-PE-Cy7 | 1:200 | Biolegend #102417 |
| PDGFRB (CD140b-APC) | 1:25 | Ebioscience #17-1402-82 |
| CD146 Brilliant Violet 421 | 1:100 | Biolegend #134709 |
| CD144 (VE-cadherin) Brilliant Violet 421 | 1:100 | Biolegend #138013 |
| CD105 Pacific Blue | 1:50 | Biolegend #120411 |
| CD29 Alexa Fluor® 488 | 1:100 | Biolegend # 102211 |
| CD45 PerCP/Cy5.5 | 1:100 | Biolegend # 103131 |
| CD73 Brilliant Violet 605™ | 1:100 | Biolegend # 127215 |
| CD146 PerCP/Cy5.5 | 1:100 | Biolegend #134709 |

**Supplementary Table 2.** Antibodies used for immunoblotting

| | Dilution | Provider |
|---|---|---|
| p38α | 1:1000 | Cell Signaling #9218 |
| phospho-p38 MAPK (Thr180/Y182) | 1:1000 | Cell Signaling #9211 |
| phospho-JNK (Thr183/Tyr185) | 1:1000 | Cell Signaling #9251S |
| JNK | 1:200 | Santa Cruz #sc-571 |
| phospho-c-jun (Ser63) | 1:1000 | Cell Signaling #9261S |
| Tubulin | 1:10000 | Sigma #T9026 |
| phospho-Smad2 (Ser465/467) | 1:1000 | Cell Signaling #3108 |
| phospho-Smad3 (Ser423/425) | 1:1000 | Cell Signaling #9520 |
| phospho-Smad3L (Ser208) | 1:500 | Elabscience #E-AB-21209 |
| ALK1 | 1:500 | Elabscience #ENT0198 |
| ALK5/TGFβ RI | 1:500 | Elabscience #ENT4627 |
| SMA | 1:5000 | Abcam # ab5694 |
| phospho-MKK3 (Ser189)/MKK6 (Ser207) | 1:1000 | Cell Signaling #9231S |
| phospho-AKT (Ser473) | 1:1000 | Cell Signaling #9271 |
| CTGF | 1:500 | Santa Cruz #14939 |
| SMAD4 | 1:200 | Santa Cruz #7966 |
| SMAD2/3 | 1:1000 | Cell Signaling #8685 |
| GAPDH | 1:1000 | Santa Cruz #20681 |
| LAMIN A/C | 1:1000 | Sigma #G8795 |
| Alexa 680 (mouse) | 1:5000 | Invitrogen #A21057 |
| Alexa 800 (mouse) | 1:5000 | Invitrogen #A20587 |
| Alexa 680 (rabbit) | 1:5000 | Invitrogen #A21076 |
| Alexa 800 (rabbit) | 1:5000 | Rockland #611131122 |
| phospho-Smad1/5 (Ser463/465) | 1:500 | Cell Signaling #41D10 |
| Smad1 | 1:1000 | Cell Signaling #D59D7 |

**Supplementary Table 3.** Antibodies used for immunohistochemistry and immunofluorescence

| | Dilution | Conditions | Provider |
|---|---|---|---|
| phospho-Smad2 (Ser465/467) | 1:500 | ON 4ºC | Cell Signalling #3108 |
| CD31 | 1:500 | 2 h RT | Abcam #28364 |
| CD68 | 1:1000 | 2 h RT | Biobyt #orb47985 |
| Ve-Cadherin | 1:200 | 2 h RT | Thermo Fisher# PA5-19612 |
| VEGF | 1:100 | 2 h RT | Millipore#ABS82 |
| SMA | 1:1000 | 30 min RT | Abcam #ab5694 |
| Endoglin (CD105) | 1:300 | 2h RT | Sino Biological #50407-rp02 |
| PDGFRB (CD140b) | 1:300 | 2 h RT | Thermo Fisher#MA5-14851 |
| CD34 | | 1 h RT | Dako #IR632 |
| GFP | 1/100 | ON 4ºC | Life Technologies #A11122 |
| eNOS | 1:100 | 2h RT | Santa Cruz# sc-376751 |
| CD146 | 1:100 | 2 h RT | Abcam# ab75769 |
| anti-goat Alexa 594 | 1/200 | 1 h RT | Life Technologies #A-11058 |
| anti-rabbit Alexa 488 | 1/200 | 1 h RT | Life Technologies #A-21441 |
| HRP conjugated anti-rabbit | | 45 min RT | ImmunoLogic #DPVR110HRP |

**Supplementary Table 4.** Primers used for PCR

| | Forward Primer (5'-3') | Reverse Primer (5'-3') |
|---|---|---|
| GAPDH | CTTCACCACCATGGAGGAGGC | GGCATGGACTGTGGTCATGAG |
| CTGF | CTGGACGGCTGCGGCTGCTG | GGTCCTTGGGCTCGTCACAC |
| Serpine-1 | GACACCCTCAGCATGTTCATC | AGGGTTGCACTAAACATGTCAG |
| TGFB1 | CTGCAAGACCATCGACATGG | GTTCCACATGTTGCTCCACA |
| TGFB2 | TTGTTGCCCTCCTACAGACTGG | GTAAAGAGGGCGAAGGCAGCAA |
| TGFB3 | GCTGTGTACGCCCCCTTTAT | AAGGGCCTGAGCAGAAGTTG |
| VEGF A | CTGTGCAGGCTGCTGTAACG | GTTCCCGAAACCCTGAGGAG |
| VE-Cadherin | AGGACAGCAACTTCACCCTCA | AACTGCCCATACTTGACCGTG |
| Tie-1 | CAAGGTCACACACACGGTGAA | GCCAGTCTAGGGTATTGAAGTAGGA |
| SMAD4 | GGGTCAGGTGCCTTAGTGAC | ACCTTTATATACGCGCTTGGGT |
| SMAD2 | GTATGGACACAGGCTCTCCG | ACCAGAATGCAGGTTCCGAG |
| SMAD3 | CTACTGCCACTTGGAGTCTCG | TCGCCCGAACTTCGCTTTTA |
| SMAD7 | AAGATCGGCTGTGGCATC | CCAACAGCGTCCTGGAGT |
| ACTA2/SMA | ATCGTCCACCGCAAATGC | AAGGAACTGGAGGCGCTG |
| Fibronectin | GTCAGTGTCTCCAGTGTCTAC | TGGCTTGCTGGCCAATCAGT |
| PDGFA | CTCTTGGAGATAGACTCCGTAG | ACTTCTCTTCCTGCGAATGG |
| Osterix | CACATCCCTGGCTGCGGCAA | CCGGGTGTGAGTGCGCACAT |
| GLUT4 | GATTCTGCTGCCCTTCTGTC | ATTGGACGCTCTCTCTCCAA |
| ANGPT1 | CATTCTTCGCTGCCATTCTG | GCACATTGCCCATGTTGAATC |
| ANGPT2 | TTAGCACAAAGGATTCGGACAAT | TTTTGTGGGTAGTACTGTCCATTCA |
| CypA | CAGACGCCACTGTCGCTTT | TGTCTTTGGAACTTTGTCTGCAA |
| PECAM | GAGCCCAATCACGTTTCAGTTT | TCCTTCCTGCTTCTTGCTAGCT |
| KDR | GCCCTGCTGTGGTCTCACTAC | CAAAGCATTGCCCATTCGAT |
| Tie-2 | ATGTGGAAGTCGAGAGGCGAT | CGAATAGCCATCCACTATTGTCC |
| FLT-1 | GAGGAGGATGAGGGTGTCTATAGGT | GTGATCAGCTCCAGGTTTGACTT |
| *Mapk14* Exon2 | GCATCGTGTGGCAGTTAAGA | GTCCTTTTGGCGTGAATGAT |
| *Mapk14* Exon12 | GCCCTCCCTCACTTCAGGAG | TGTGCTCGGCACTGGAGACC |
