# Supplementary material for: Regulation of tumor angiogenesis and mesenchymal–endothelial transition by p38α through TGF-β and JNK signaling
Source: Nat Commun. 2019 Jul 11;10:3071. doi: 10.1038/s41467-019-10946-y (PMC6624205; doi:10.1038/s41467-019-10946-y)
Supplement: Supplementary file 5 — Reporting Summary [file 41467_2019_10946_MOESM5_ESM.pdf]

nature research

Corresponding author(s): Angel R. Nebreda

Last updated by author(s): Jun 4, 2019

Reporting Summary

Nature Research wishes to improve the reproducibility of the work that we publish. This form provides structure for consistency and transparency in reporting. For further information on Nature Research policies, see Authors & Referees and the Editorial Policy Checklist.

Statistics

For all statistical analyses, confirm that the following items are present in the figure legend, table legend, main text, or Methods section.

n/a	Confirmed	
☐	☒	The exact sample size (*n*) for each experimental group/condition, given as a discrete number and unit of measurement
☐	☒	A statement on whether measurements were taken from distinct samples or whether the same sample was measured repeatedly
☐	☒	The statistical test(s) used AND whether they are one- or two-sided<br>*Only common tests should be described solely by name; describe more complex techniques in the Methods section.*
☐	☒	A description of all covariates tested
☐	☒	A description of any assumptions or corrections, such as tests of normality and adjustment for multiple comparisons
☐	☒	A full description of the statistical parameters including central tendency (e.g. means) or other basic estimates (e.g. regression coefficient) AND variation (e.g. standard deviation) or associated estimates of uncertainty (e.g. confidence intervals)
☐	☒	For null hypothesis testing, the test statistic (e.g. *F*, *t*, *r*) with confidence intervals, effect sizes, degrees of freedom and *P* value noted<br>*Give P values as exact values whenever suitable.*
☒	☐	For Bayesian analysis, information on the choice of priors and Markov chain Monte Carlo settings
☒	☐	For hierarchical and complex designs, identification of the appropriate level for tests and full reporting of outcomes
☒	☐	Estimates of effect sizes (e.g. Cohen's *d*, Pearson's *r*), indicating how they were calculated

Our web collection on statistics for biologists contains articles on many of the points above.

Software and code

Policy information about availability of computer code

Data collection	Cells populations from colon tissue or tumors were capture using BD FACSDiva™ software v6.1.3<br>Immunoblotting were visualized using Odyssey Infrared Imaging System (Li-Cor, Biosciences).<br>Microarrays were performed using a GeneAtlas Fluidics station and scanner (Affymetrix).<br>The immunofluorescence samples were visualized with Confocal Microscope Leica SPE or Zeiss LSM.<br>qRT-PCR was performed in a Bio-Rad C1000 thermal cycler machine
Data analysis	Stadistical analysis were performed using GraphPad Prism 7 software<br>Cells populations were analyzed using BD FACSDiva™ software v6.1.3 and FlowJo<br>Immunohistochemistry quantifications were performed using ImageJ-Fiji macro. See Code availability.<br>Immunoblotting quantification was performed using ImageJ-Fiji and Odyssey Infrared Imaging System (Li-Cor, Biosciences)<br>Angiogenesis assays were quantified using an ImageJ-Fiji macro (http://image.bio.methods.free.fr/ImageJ/?Angiogenesis-Analyzer-for-ImageJ&lang=en).

For manuscripts utilizing custom algorithms or software that are central to the research but not yet described in published literature, software must be made available to editors/reviewers. We strongly encourage code deposition in a community repository (e.g. GitHub). See the Nature Research guidelines for submitting code & software for further information.

Data

Policy information about availability of data

All manuscripts must include a data availability statement. This statement should provide the following information, where applicable:

- Accession codes, unique identifiers, or web links for publicly available datasets
- A list of figures that have associated raw data
- A description of any restrictions on data availability

Microarrays primary accession codes GSE83810.
The following link is exclusively for the reviewers use:

https://www.ncbi.nlm.nih.gov/geo/query/acc.cgi?token=gvudgegoblqztkv&acc=GSE83810
Cell population profiles from human colorectal tumors were derived from two datasets GEO repository: GSE39395 and GSE39396A.
All figures have associated raw data.

Field-specific reporting

Please select the one below that is the best fit for your research. If you are not sure, read the appropriate sections before making your selection.

☒ Life sciences ☐ Behavioural & social sciences ☐ Ecological, evolutionary & environmental sciences

For a reference copy of the document with all sections, see nature.com/documents/nr-reporting-summary-flat.pdf

Life sciences study design

All studies must disclose on these points even when the disclosure is negative.

Sample size	Sample size was based on literature-reported experiments and previous experience in our group using similar strains and treatments.
Data exclusions	Premature death following AOM/DSS treatment was a criteria for exclusion. The remaining animals were all included in the studies. Criteria was pre-established.
Replication	Experimental findings were reliably reproduced. Experiments were performed at least three times.
Randomization	Experimental animals were usually 8-weeks old and groups were balanced in terms of sex and weight.
Blinding	Animals of different genotypes were caged together and were all treated in the same way. The investigator could not distinguish them during the treatments or when assessing outcomes. To be included in Methods

Reporting for specific materials, systems and methods

We require information from authors about some types of materials, experimental systems and methods used in many studies. Here, indicate whether each material, system or method listed is relevant to your study. If you are not sure if a list item applies to your research, read the appropriate section before selecting a response.

Materials & experimental systems

n/a	Involved in the study	
☐	☒	Antibodies
☐	☒	Eukaryotic cell lines
☒	☐	Palaeontology
☐	☒	Animals and other organisms
☒	☐	Human research participants
☒	☐	Clinical data

Methods

n/a	Involved in the study	
☒	☐	ChIP-seq
☐	☒	Flow cytometry
☒	☐	MRI-based neuroimaging

Antibodies

Antibodies used	Described in Supplementary Tables 1, 2 and 3
Validation	Validation statement for each western-blot primary antibody is provided on the manufacture's website.<br>For flow cytometry and immunofluorescence, antibodies were validated as noted on manufacturer's website, and most of antibodies specificity was confirmed in the literature. In addition, the stainings were consistent with the predicted cellular localization of the protein.<br>Antibodies were further validated by using positive and negative controls in our studies.

Eukaryotic cell lines

Policy information about cell lines

Cell line source(s)	HT-29 and CMT93 cancer cell lines were purchased from ATCC. H5V murine endothelial cells were provided by Raul Mendez's lab at IRB Barcelona.<br>MSCs and perivascular cells were isolated from p38a floxed Ub-CRE-ERT2 mice and processed as described in Supplementary information.
Authentication	MSC identity was confirmed as described in Supplementary information

Mycoplasma contamination	Cell lines were tested weekly for mycoplasma contamination.
Commonly misidentified lines (See ICLAC register)	None of the cell lines used were in the list of commonly misidentified cell lines.

Animals and other organisms

Policy information about studies involving animals; ARRIVE guidelines recommended for reporting animal research

Laboratory animals	Included in Methods<br>Floxed allele of Mapk14 encoding p38α was combined with the UBC-Cre-ERT2, FSP1-Cre, PDGFRB-Cre-ERT2 and with the linage reporter Tomato/GFP inserted into the Rosa26 locus. Experimental animals (males and females) were usually 8-weeks old and groups were balanced in terms of sex and weight.
Wild animals	Study did not involve wild animals
Field-collected samples	Study did not involve samples collected from the field.
Ethics oversight	Mice were housed according to national and European Union regulations, and protocols were approved by the animal care and use committee of Barcelona Science Park (CEEA-PCB)

Note that full information on the approval of the study protocol must also be provided in the manuscript.

Flow Cytometry

Plots

Confirm that:

☒ The axis labels state the marker and fluorochrome used (e.g. CD4-FITC).

☒ The axis scales are clearly visible. Include numbers along axes only for bottom left plot of group (a 'group' is an analysis of identical markers).

☒ All plots are contour plots with outliers or pseudocolor plots.

☒ A numerical value for number of cells or percentage (with statistics) is provided.

Methodology

Sample preparation	Colons were dissected, opened longitudinally and washed with cold PBS, incubated with 8 mM EDTA at 37ºC for 15 min. Supernatants were centrifuged at 1200 rpm for 5 min at 4ºC, and pelleted cells were digested with Dispase II (0.5 mg/ml) at 37ºC for 25 min to isolated epithelial cells. To obtain lamina propria (mesenchymal and leucocytes cells) colon pieces after EDTA incubations were collected, cut into small pieces (2-3 mm) and digested with mix of collagenase A (1.75 mg/ml) at 37ºC for 45 min. To quantify different cell populations, single cell suspensions from both purifications were co-stained with pan-leukocyte antigen CD31-PE-Cy7 for endothelial cells or PDGFRB-APC and CD146 PerCP/Cy5.5 for mesenchymal/perivascular cells, and analyzed on FACS Aria 2.0 (BD Biosciences).
Instrument	FACS Aria 2.0 (BD Biosciences)
Software	FlowJo v 10<br>BD FACSDiva™ software v6.1.3
Cell population abundance	Perivascular cells were sorted using PDGFRB+ and CD146+ antibodies from colon tissue of UBC-Cre-ERT2 mice. In Supplementary Figure 6e we show the Flow cytometry scheme indicating the approach to isolate perivascular cells. The abundance of PDGFRB+ and CD146+ cells for these samples could not be assessed.
Gating strategy	Cells were selected in the forward scatter/side scatter (FSC/SSC) dot plot and then gated to exclude cellular aggregates in the FSC/FSC dot plot. Gates for GFP and Tomato cells were set to compare the expression of endothelial and perivascular markers in different conditions. As negative control, a sample with no detectable fluorochrome expression was used.

☒ Tick this box to confirm that a figure exemplifying the gating strategy is provided in the Supplementary Information.
