# Supplementary material for: Regulation of tumor angiogenesis and mesenchymal–endothelial transition by p38α through TGF-β and JNK signaling
Source: Nat Commun. 2019 Jul 11;10:3071. doi: 10.1038/s41467-019-10946-y (PMC6624205; doi:10.1038/s41467-019-10946-y)
Supplement: Supplementary file 6 — Description of Additional Supplementary Files [file 41467_2019_10946_MOESM6_ESM.docx]

**Title:** Supplementary Movie 1.
**Description:** Time-lapse of orthogonal projections along the vertical axis showing central lumen in immortalized p38aΔ MSCs cultured in a 3D collagen gel.

**Title:** Supplementary Movie 2.
**Description:** Time-lapse of orthogonal projections along the vertical axis showing central lumen in primary p38aΔ MSCs cultured in a 3D collagen gel.
